# Nickel—Alumina Catalysts for the Transformation of Vegetable Oils into Green Diesel: The Role of Preparation Method, Activation Temperature, and Reaction Conditions

**DOI:** 10.3390/nano13030616

**Published:** 2023-02-03

**Authors:** Ioannis Nikolopoulos, George Kogkos, Vasiliki D. Tsavatopoulou, Eleana Kordouli, Kyriakos Bourikas, Christos Kordulis, Alexis Lycourghiotis

**Affiliations:** 1 Department of Chemistry, University of Patras, GR-26504 Patras, Greece; 2 School of Science and Technology, Hellenic Open University, Parodos Aristotelous 18, GR-26335 Patras, Greece; 3 Foundation for Research and Technology, Institute of Chemical Engineering Science (FORTH/ICE-HT), Stadiou Str., Platani, P.O. Box 1414, GR-26500 Patras, Greece

**Keywords:** Ni catalyst, vegetable oil hydrodeoxygenation, green diesel, renewable diesel, biofuel

## Abstract

Two nickel alumina catalysts containing 60 wt. % Ni were synthesized by wet impregnation and co-precipitation in order to study the effect of preparation methods on the catalytic efficiency concerning the transformation of sunflower oil into green diesel. The effect of activation temperature on the catalytic efficiency of the most active catalyst was also studied. The catalysts were characterized using various techniques and which were evaluated in the aforementioned reaction using a semi-batch reactor. The catalyst prepared by co-precipitation exhibited a higher specific surface area and smaller mean crystal size of the nickel nanoparticle (higher nickel metallic surface). These justify its higher efficiency with respect to the corresponding catalyst synthesized by wet impregnation. The increase in the activation temperature from 400 to 600 °C increased the size of the nickel nanoparticles through sintering, thus destroying the small pores. These led to a decrease in the nickel surface and specific surface area and, thus, to a decrease in the catalytic efficiency. The optimization of the reaction conditions over the most active catalyst (prepared by co-precipitation and activated at 400 °C) leads to the complete transformation not only of the sunflower oil (edible oil) but also of waste cooking oil (non-edible oil) into green diesel. The liquid produced after the hydrotreatment for these two feedstocks for 7 h, at H_2_ pressure 40 bar and temperature 350 °C using 100 mL of oil and 1 g of catalyst was composed of 97 and 96 wt. % of green diesel, respectively.

## 1. Introduction

The gradual replacement of petrol diesel by the so-called green diesel is significant for confronting carbon dioxide emissions that are implicated in global warming as well as for the increasing depletion of fossil fuels [1]. The production of green diesel can be obtained via the catalytic hydrotreatment of fresh and used plant oils and animal fat [2,3]. Upon this treatment, the triglycerides contained in the biomass are transformed into hydrocarbons. Noble metals (mainly Pd/C) [4] and the conventional NiMo/γ-Al_2_O_3_ sulfided catalysts [5,6] were first studied. The high cost of the noble metals and the necessity to add sulfur to the feedstock to keep constant the catalytic performance of the NiMo/γ-Al_2_O_3_ sulfided catalysts, which might contaminate the final product, have turned research interests to cheaper and quite efficient nickel catalysts. We have reviewed the domain concerning nickel catalysts from 2016 [7]. This research has been mainly focused on metallic catalysts. In this context, the research deals with nickel loadings, supports, preparation methods, and promoters, as well as with the mechanism of the process. Intersecting reports concern nickel phosphides, carbides, and nitrides. Since then and during the last years, the investigation has continued intensively on the aforementioned issues [8,9,10,11,12,13,14,15,16,17,18,19]. These studies have shown that the selective deoxygenation (SDO) of triglycerides (very small extent fragmentation of the side chains of triglycerides) results in n-alkanes in the diesel range (n-C_15_–n-C_18_) which can be achieved at 240–360 °C and a hydrogen pressure of 10–80 bar [7]. They also suggested a detailed SDO mechanism, which involves parallel and consecutive reactions, e.g., hydrogenolysis, hydrogenation, hydrodeoxygenation (HDO), decarboxylation/decarbonylation (deCOx), and dehydration, but no cracking [7,20].

Our group has contributed to the domain by developing nickel-alumina [21,22] and nickel-zirconia catalysts [23]. The nickel-alumina catalysts were synthesized by co-precipitation, which is considered quite convenient for preparing catalysts with a highly active surface, namely catalysts with relatively small nickel nanoparticles, even for high nickel loading. In particular, the study of a series of nickel-alumina co-predicated catalysts with varying nickel loading (0–100 wt. %) showed that the highest active surface and, thus, the highest catalytic efficiency is obtained over the catalyst containing 60 wt. % Ni. Based on this catalyst, copper [24], zinc [25], molybdenum oxide [26,27], and tungsten oxide [28] promoted nickel-alumina co-precipitation catalysts which were then synthesized. These highly active metal density catalysts were successfully tested in the SDO of sunflower oil (SFO) and waste cooking oil (WCO) at 310 °C and 40 bar hydrogen pressure under free solvent conditions and a very high feedstock volume to catalyst mass ratio (100 mL/1 g).

In the present work, we are returning to the un-promoted nickel-alumina catalyst containing 60 wt. % Ni in order to compare the co-precipitation method followed previously for its preparation to the wet impregnation method and usually followed for the synthesis of nickel-supported catalysts [29,30]. Moreover, we studied the effect of activation temperature on the physicochemical properties and the catalytic efficiency of the most active catalyst. Finally, we attempted to achieve a complete SFO transformation into green diesel by increasing the reaction temperature. The appropriate reaction temperature to be determined can also be used for the SDO of WCO to green diesel.

## 2. Materials and Methods

Feedstocks

The SFO used was purchased from the local market. Most side chains of natural triglycerides involved in the SFO corresponded to linoleic (71.7%), oleic (15.9%), palmitic (5.8%), and stearic acid (3.9%) [21]. The SFO was chosen as a feedstock because it is currently used in Greece and other European countries for producing biodiesel, and therefore, it is expected to be used for producing green diesel in the near future. Several studies have demonstrated that the production of green diesel is of a lower or equal cost compared to biodiesel production [31,32,33]. Furthermore, although sunflower is an edible oil crop, genetically modified sunflower grown on marginal land has been recognized as one of the most sustainable biofuel sources [34]. This is because it does not demand arable lands. It should be noted that genetically modified sunflower seeds contain about 50% oil whereas the quality of this oil does not satisfy the food standards [34]. Collect Oil Company, which collects used oils from local restaurants and residences, kindly offered this to the WCO. Important specifications of WCO have been given elsewhere [22]. In brief, it has 0.83% acidity expressed as oleic acid, 0.14 wt. % water, small amounts of mono- and di-glycerides, stearic acid, and bulky compounds (starch and proteins). It should be noted that the price today of WCO is approximately equal to ¼ of that of SFO. The economics of green diesel production strongly depends on the capacity of production units and the price of feedstock [33].

Catalyst synthesis

Nickel nitrate (Ni(NO_3_)_2_·6H_2_O, Alpha Aesar, Ward Hill, Shrewsbury, MA, USA) and commercial γ-alumina (Akzo, particle size smaller than 150 nm) were calcined at 500 °C, and distilled water was used for the catalyst prepared by wet impregnation. A rotary evaporator (BUCHI Rotavapor R-114) was used involving a water bath, spherical flask, condenser, thermometer, vacuum pump, and vacuum regulator. A proper amount of the nitrate salt was dissolved in 150 mL of distilled water inside the spherical flask, and then a proper number of γ-alumina particles was added into the spherical flask. The suspension was stirred for 30 min by rotating the spherical flask to homogenize, and then the temperature and pressure were adjusted at 50 °C and 55 mbar, respectively. The suspension remained under stirring for about 20 min, which was sufficient time to obtain a dry solid.

Nickel nitrate [Ni(NO_3_)_2_·6H_2_O (Merck, kGaA, Darmstadt, Germany)] and aluminum nitrate [Al(NO_3_)_3_·9H2O, Alfa Aesar] were used for the catalyst prepared by co-precipitation. Proper amounts of these substances were dissolved in distilled water, and the solution was fed, using a syringe pump, to a beaker containing an ammonium hydroxide aqueous solution (pH = 8). A pH-stat (Metrohom-645, iso-Dosimat) was used, which fed the beaker with an ammonium hydroxide aqueous solution (25–28% PENTA) for maintaining a constant pH. A suspension of nickel and aluminum hydroxides was produced. The suspension was vacuum filtered; the precipitate obtained was washed with distilled water and air dried at 110 °C overnight. A similar procedure was followed for the preparation of alumina by precipitation using only an aluminum nitrate aqueous solution. 

Catalyst activation

The activation procedure of the dried samples involved two steps. In the first step, each sample was placed in a fixed-bed quartz reactor. The temperature was increased from room temperature up to 400 °C (5 °C/min) under the flow of argon (35 mL/min). The sample remained at this temperature for 1 h. In this period, the nickel salts (or hydroxides) in the sample prepared by wet impregnation and the nickel and aluminum hydroxides in the samples prepared by precipitation were decomposed to their corresponding oxides. In the second step, the oxide precursor samples were reduced at various temperatures (400, 500, and 600 °C) for 2.5 h by switching the Ar stream to H_2_ (30 mL min^−1^), and then the reduced catalyst that was obtained was cooled down to an ambient temperature under Ar (35 mL min^−1^). Activation was completed by passivating the reduced catalyst and feeding the reactor with 1 *v*/*v*% O_2_ in Ar (20 mL min^−1^) for 0.5 h. 

Symbolisms

The commercial γ-alumina and the alumina prepared by precipitation were symbolized by 0NiAl_wi_ and 0NiAl_cp_, respectively. The catalysts prepared by wet impregnation and co-precipitation and activated at 400 °C were, respectively, symbolized by 60NiAl_wi(400)_ and 60NiAl_cp(400)_. Finally, the catalysts prepared by co-precipitation were activated at 500 °C and 600 °C, respectively, as symbolized by 60NiAl_cp(500)_ and 60NiAl_cp(600)_.

Characterization methods

SEM–EDS (SEMJEOL JSM6300 equipped with an Energy Dispersive Spectrometry accessory) was used to examine the morphology and measure the nickel loading of the prepared catalysts. The latter was also confirmed by atomic adsorption spectroscopy (AAS—Perkin Elmer AAnalyst 700) after the dissolution of the prepared catalysts with “aqua-regia”.

Nitrogen adsorption–desorption isotherms were obtained using a Micromeritics apparatus (Tristar 3000 porosimeter) which was elaborated to determine the pore volume distribution, the BET specific surface area, the BJH specific pore volume, and the BJH mean pore diameter of the activated samples.

The identification of the crystal phases presented in the activated catalysts while the calculation of the mean crystal diameter of the metallic nickel nanoparticles was achieved by means of X-ray diffraction patterns recorded in the 2θ range 20–80° using a Brucker D8 Advance diffractometer equipped with a nickel-filtered CuKα (1.5418Å) radiation source.

To obtain the H_2_-TPR profiles of the precursor catalysts, a weighted amount (50 mg) of them was placed in a quartz micro-reactor. The hydrogen–argon mixture (H_2_/Ar: 5/95 *v*/*v*) was passed through the reactor (40 mL/min), and the temperature was raised from room temperature up to 1000 °C with a rate equal to 10 °C min^−1^. The reduction in the sample is reflected by the decrease in hydrogen concentration in the mixture. This was monitored in the reactor outlet by a thermal conductivity detector (TCD). A cold trap placed before TCD was used to trap the water released. 

The same setup was used for obtaining the NH_3_-TPD profiles in order to determine the acid site distribution. An amount of the activated catalyst (100 mg) was placed in the reactor, and helium was fed (30 mL min^−1^) for 30 min to remove the adsorbed species. A stream of ammonia was fed to the reactor for 30 min at room temperature. Then, it was replaced by a helium stream for removing the physisorbed ammonia. The progressive desorption of chemisorbed ammonia was achieved by increasing the temperature linearly (10 °C min^−1^) up to 700 °C. The amount of desorbed ammonia was monitored in the reactor outlet by a TCD detector. 

The aforementioned setup was also used for CO-chemisorption measurements following the two-cycle pulse technique at 30 °C using 100 mg of the activated catalyst. The amount of chemisorbed CO was calculated by subtracting the CO adsorbed during the second cycle from that of the first one. Details concerning the catalyst characterization have been reported in our previous papers [21,22,23,24,25,26,27,28,35]. 

The total carbon (TC) content of the used catalysts was determined by the high temperature - dry combustion technique using a Shimadzu TOC-L_CSH_ main unit and SSM-5000A sample combustion unit [Shimadzu, Kyoto, Japan]. The CO_2_ formed is subsequently transferred to an NDIR detector. The weighted sample was inserted into the TC furnace of the instrument and combusted at 900 °C in a stream of oxygen. The Cobalt/Platinum mixed catalyst was used to ensure and accelerate combustion. The generated gases were then passed through a drain vessel, and a halogen scrubber removed the vapors and halogenated the compounds respectively. The carbon dioxide was measured by the NDIR detector and was finally trapped/removed by the absorption of soda.

Catalysts evaluation

The catalysts were evaluated using a semi-batch reactor (300 mL, Autoclave Engineers). The catalytic tests were performed at various temperatures in the range 310–350 °C, with an SFO or WCO volume to catalyst mass ratio equal to 100 mL g^−1^, keeping the hydrogen pressure and flow constant at 40 bar and 100 mL/min, respectively. Each reaction run was monitored for 9 h. Liquid samples were taken from the reactor every hour. and their composition was determined by gas chromatography. A gas chromatograph (Shimadzu GC-2010 Plus) equipped with a flame ionization detector and a column (SUPELCO, MET-Biodiesel, l = 14 m, d = 0.53 mm, tf = 0.16 μm) were used. Gas chromatography-mass spectrometry (Shimadzu GCMS-QP2010 ultra) was also used for the identification of the compounds in the liquid samples. All the tests were performed at least three times, and the conversion and the yield values that were obtained differed by less than 2%. Blank experiments performed without a catalyst or using the corresponding supports did not produce any hydrocarbon. More details have been reported in our previous articles [21,22,23,24,25,26,27,28,35]. 

## 3. Results and Discussion

### 3.1. Effect of Preparation Method and Activation Temperature on the Porosity and Structure of the Catalysts Studied 

**Figure 1:**
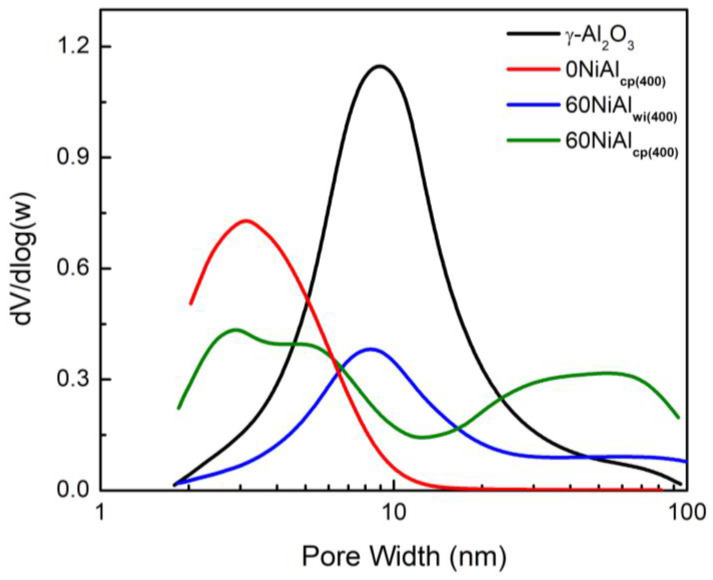
Pore volume distributions of aluminas and nickel–alumina catalysts synthesized using different methods.

In the present work nickel–alumina catalysts with high Ni loading (60 wt.%) were prepared by two different methods (wet impregnation and co-precipitation). SEM-EDS and AAS analyses of final catalysts confirmed the Ni loading, while the SEM images presented in Figure S1 revealed that the co-precipitation method led to a material that was more porous. N_2_ adsorption—desorption isotherms of the studied samples are presented in Supplementary Material (Figures S2 and S3). Based on these isotherms, the effect of the preparation method on the pore volume distribution of aluminas and nickel–alumina catalysts is determined and illustrated in Figure 1. The values of the most important physicochemical parameters of the solids studied are compiled in Table 1. 

An inspection of Figure 1 shows that the commercial alumina (γ-Al_2_O_3_) presented a single-peak distribution centered at 9 nm. This justifies the relatively large specific surface area of this material (260 m^2^·g^−1^) as well as the value of the average pore diameter (6.6 nm). The catalyst prepared by wet impregnation using this support (60NiAl_wi(400)_) presents a similar distribution curve suggesting that the rigid pore structure of the pre-existing γ-alumina was not affected markedly upon impregnation. However, the volume of the pores with a diameter in the range of 2–40 nm is considerably smaller, most probably due to the partial blocking of these pores by nickel phases located inside or on the mouths of them.

This is in line with the much lower value of the specific surface area (111 m^2^·g^−1^) and the specific pore volume (0.29 cm^3^·g^−1^) determined for this catalyst with respect to the support (Table 1). The slight increase in the mean pore diameter from 6.6 to 7.6 nm is due to the appearance of additional pores in the range 40–100 nm. 

The alumina prepared by precipitation using ammonia as a precipitating agent (0NiAl_cp(400)_) presents mainly a mesoporous structure and single-peak distribution in a low pore width range (<15 nm) centered at 3 nm. This justifies the very large specific surface area of this alumina (344 m^2^·g^−1^) as well as the small value for the average pore diameter (3.2 nm) (Table 1). In contrast, the nickel–alumina catalyst prepared by the same method (60NiAl_cp(400)_) exhibits a more complicated pore volume distribution curve with four maxima at about 2.5, 5.5, 30, and 60 nm. This indicates the critical role of nickel species that are present in various preparation steps in the determination of the pore structure of the mixed solid. The decrease in the population of the pores with a diameter <7.0 nm is in line with the decrease in the specific surface area from 344 to 272 m^2^·g^−1^, whereas the increase in the pore population with a diameter >7.0 nm is responsible for the increase in the specific pore volume from 0.34 to 0.60 cm^3^·g^−1^ and the average pore diameter from 3.2 to 6.6 nm (Table 1). Overall, the preparation method influences the pore structure of the nickel–alumina catalysts. The catalyst prepared by co-precipitation exhibited more promising textural properties compared to that prepared by wet impregnation. 

**Figure 2:**
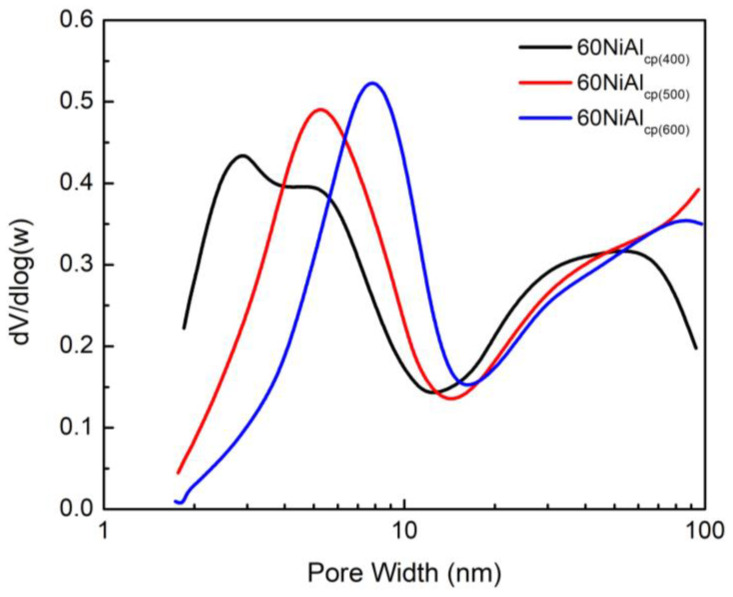
The effect of activation temperature on the pore volume distribution of the catalyst synthesized by co-precipitation.

The most promising catalyst (60NiAl_cp(400)_) was further studied in order to investigate the effect of activation temperature on porosity (Figure 2). The inspection of this figure shows that the four–peak curve described before for the sample activated at 400 °C was transformed into three-peak curves after activation at 500 and 600 °C as the low pore width peak (2.5 nm) disappeared. The intensity of the peak with a maximum of 5.5 nm increased for the sample 60NiAl_cp(500)_ and increased further at 8.0 nm for the sample 60NiAl_cp(600)_. A continuous increase in the pore volume was observed in the range of 20–100 nm. The observed changes of 60NiAl_cp(400)_ catalyst porosity as the activation temperature increased indicate that its small pores collapsed, presumably due to the increasing sintering of the nickel nanoparticles(see below). The above justifies the progressive decrease in the specific surface area from 272 to 193 and then to 143 m^2^·g^−1^, as well as the progressive increase in the average pore diameter from 6.6 to 8.7 and then to 10.9 nm (Table 1). In summary, the increase in the activation temperature affected the catalysts’ porosity considerably. The catalyst activated at 400 °C seemed to exhibit the most promising textural properties. 

**Figure 3:**
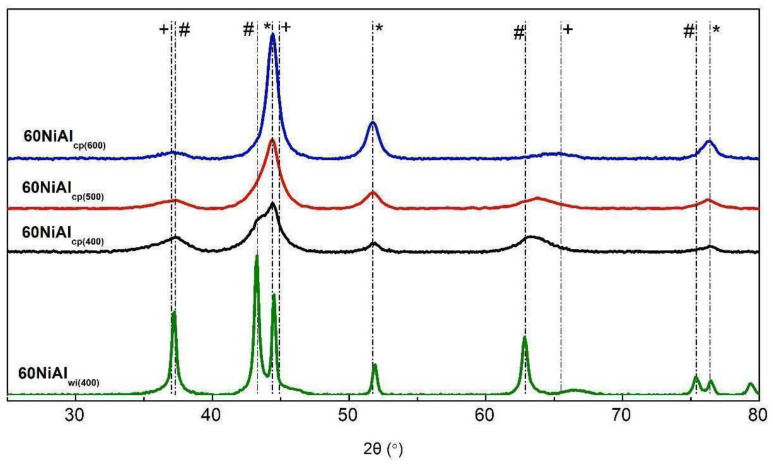
XRD patterns of the catalysts studied. (*): Ni^0^, (#): NiO, (+): NiAl_2_O_4_.

Figure 3 illustrates the XRD patterns of the catalysts studied. Peaks due to the Ni^0^ [2θ: 44.6° (111), 51.8° (200), 76.3° (220), JCPDS 04-0850], NiO [2θ: 43.3° (200), 37.3° (111), 62.8° (220), 75.4^o^, JCPDS 22–1189], and NiAl_2_O_4_ [2θ: 37.0° (311), 45.0° (400), 65.4° (440), JCPDS 10-0339] crystals were identified. The XRD patterns of the precipitated alumina (0NiAl_cp(400)_) and the commercial support (0NiAl_wi(400)_) are illustrated in Figure S4. An inspection of this figure reveals that the 0NiAl_cp(400)_ sample is almost amorphous, justifying its large specific surface area (Table 1).

A comparison of the patterns for the samples activated at 400 °C shows that the peaks due to Ni^0^ and NiO are much sharper for the sample 60NiAl_wi(400),_ suggesting the formation of larger nanocrystals. In fact, the mean crystal size of the Ni^0^ calculated for this sample was found to be equal to 20.2 nm, whereas that for the 60NiAl_cp(400)_ sample was smaller than 4 nm (Table 1). This finding indicates that the co-precipitation methodology results in a catalyst with enhanced Ni dispersion and, thus, a higher Ni^0^ surface area than that of the wet-impregnated catalyst. This was confirmed both by calculations based on the average crystallite size and by measurements of the amount of chemisorbed carbon monoxide (Table 1) and is compatible with the influence of the preparation method on the textural properties described before. 

A comparison of the XRD patterns of the samples synthesized by co-precipitation and activated at various temperatures shows that the increase in the activation temperature brought about the transformation of more and more NiO into Ni^0^. This was deduced by a progressive decrease in the relative magnitude of the peaks at 2θ, which was equal to 43.3, 37.3, and 62.8° as the activation temperature increased. This is beneficial from the catalytic point of view, as the active sites for the catalytic transformation of SFO into green diesel were located on the surface of Ni^0^ nanoparticles [21].

In contrast, the increase in the activation temperature was brought about by an increase in the size of the supported nickel nanoparticles, which was deduced by the progressive increase in the sharpness of the peaks due to the Ni^0^ with the activation temperature (see peaks at 2θ: 44.6, 51.8, and 76.3^o^). In fact, the mean crystal diameter of the Ni^0^ nanoparticles increased from <4 to 7.6 and then to 8.2 nm with the activation temperature (Table 1). CO chemisorption results also confirmed the decrease in the Ni^0^ surface area with the increase in activation temperature (Table 1). This effect, compatible with textural results, is unfavorable from the catalytic point of view as it is related to the progressive decrease in the surface of metallic nickel (Table 1) calculated on the base of the mean crystal diameter.

**Figure 4:**
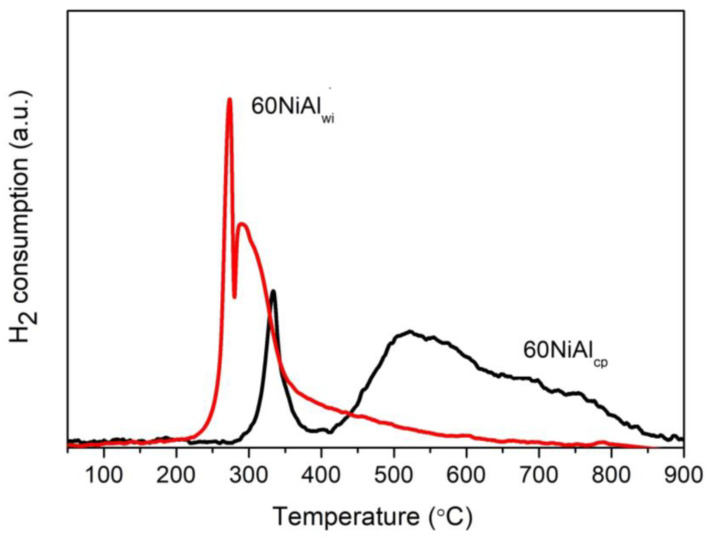
H_2_-TPR profiles of precursor catalysts prepared by wet impregnation (60NiAl_wi_) and co-precipitation (60NiAl_cp_) thermally treated at 400 °C under Ar.

Figure 4 shows H_2_-TPR profiles of oxide precursor samples (thermally treated at 400 °C under Ar) prepared by wet impregnation (60NiAl_wi_) and co-precipitation (60NiAl_cp_). The 60NiAl_wi_ precursor sample was easily reduced, appearing as two reduction peaks with maxima at 275 and 290 °C and a wide shoulder zeroing at about 700 °C. This profile indicates that the main fraction of NiO interacts weakly with γ-Al_2_O_3_, while a smaller part interacts moderately with it [36,37]. However, the XRD results (Figure 3) showed that the corresponding catalyst activated at 400 °C contained a significant amount of NiO, and one should expect that this NiO would be reduced at higher temperatures, but according to H_2_-TPR results (Figure 4), this was not the case. This presumably indicates that the NiO remaining after reduction at 400 °C is encapsulated in clogged pores of γ-Al_2_O_3,_ and thus, its reduction was hindered. This explanation is in good agreement with the drastic change in 60NiAl_wi(400)_ catalyst porosity in comparison to γ-Al_2_O_3,_ which was used for its synthesis (Table 1). An alternative explanation could be that the superficial reduced metallic nickel (reduced too early) blocked a reduction in the other portion of NiO located more inside a bulk of NiO. So, a kind of eggshell of metallic Ni prevents the reduction of NiO in the egg yolk. One can assume that the impregnation method created large crystals of NiO, which is in agreement with the XRD results (Figure 3). 

The H_2_-TPR profile of the 60NiAl_cp_ sample has two reduction temperature regions. The reduction peak at 330 °C is attributed to NiO weakly interacting with Al_2_O_3_. The wide reduction peak extended from 410 up to 880 °C shows, in addition, the formation of various NiO-species, which interact stronger with Al_2_O_3_ forming non-stoichiometric surface aluminates (NiAlxOy) that are highly dispersed on the surface of the support, and stoichiometric NiAl_2_O_4_ [37]. Finally, we have to note that the hydrogen consumption in the 60NiAl_cp_ sample was found to be 42% higher than that in the 60NiAl_wi_. This finding confirms our previous conclusions that (i) in the latter sample, a part of NiO was encapsulated in clogged pores of γ-Al_2_O_3_ and (ii) in the co-precipitated catalysts, a higher dispersion of the Ni precursor phase was achieved, which finally resulted in a higher active surface (Table 1).

**Figure 5:**
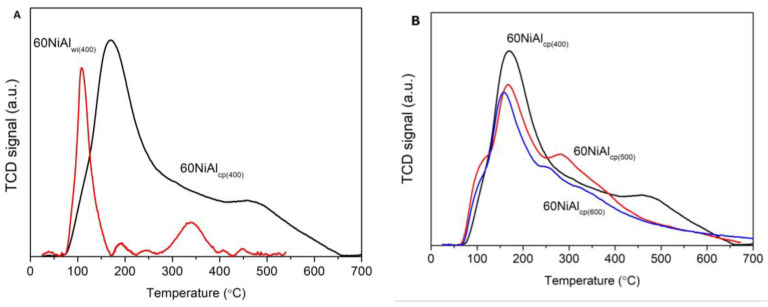
Influence of preparation method (**A**) and activation temperature (**B**) on NH_3_-TPD profiles of Ni-Al_2_O_3_ catalysts containing 60 wt. % Ni.

The acidity of the catalysts was determined by NH_3_-TPD. Figure 5 presents the corresponding curves. These curves allowed us to calculate the total acidity values of the catalysts (see Table 1). These values show that the catalyst prepared by co–precipitation had a higher acid site population than that of the catalyst prepared by wet impregnation. An inspection of the corresponding curve shows that the 60NiAl_wi(400)_ catalyst exhibits only weak (desorption temperature: <250 °C) and moderate (desorption temperature: 250–450 °C) acid sites, while the 60NiAl_cp(400)_ catalyst exhibits, in addition, strong acid sites (desorption temperature: >450 °C) (Figure 5A). On the other hand, Figure 5B shows that the increase in the activation temperature results in a gradual decrease in the acid site population (Table 1). This decrease concerns all kinds of acid sides. It is well known that acid sites play an important role in the mechanism of the triglyceride SDO [12,38]. Papanikolaou et al. [12], working on Ni/zeo-type catalysts, revealed that the proximity between the acidic and the hydrogenation functions led to catalysts with high selectivity to diesel range products enhancing both deCOx and HDO pathways.

Overall, the N_2_-physisorption, XRD, H_2_-TPR, and NH_3_-TPD results show that the catalysts prepared by co-precipitation exhibit physicochemical characteristics that are more promising for the SDO of triglyceride biomass than the catalyst prepared by wet impregnation. These characteristics are even better for the co-precipitated sample activated at 400 °C.

### 3.2. Effect of Preparation Method and Activation Temperature on the Catalytic Efficiency of the Catalysts Studied

**Figure 6:**
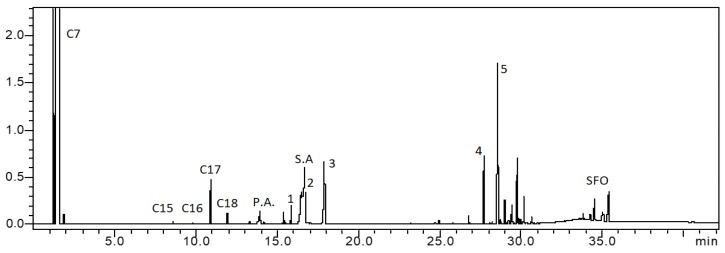
Typical chromatogram recorded for reaction time 9 h over the catalyst 60NiAl_cp(500)_ taken as an example. C7: solvent for the dilution of the liquid sample, n-C15, n-C16,n-C17,n-C18: n-alkanes, P.A.: palmitic acid, S.A.: stearic acid, 1,2,3,4,5: 1-octadecanol, methyl stearate, propyl stearate, palmityl stearate, stearyl stearate.

Figure 6 illustrates a typical chromatogram that was recorded after sampling the liquid phase of the reactor for a reaction time of 9 h over the sample 60NiAl_cp(500),_ taken as an example.

An inspection of this figure shows that the molecules identified in the liquid phase upon the SDO of SFO over the catalysts studied were n-alkanes (n-C_15_–n-C_18_), stearic and palmitic acid, propyl, methyl, palmityl, and stearyl stearate, as well as very small amounts of 1-decaoctanol and unreacted SFO. The aforementioned products are compatible with the well-established SDO mechanism over the nickel-reduced catalysts mentioned in the Introduction.

**Figure 7:**
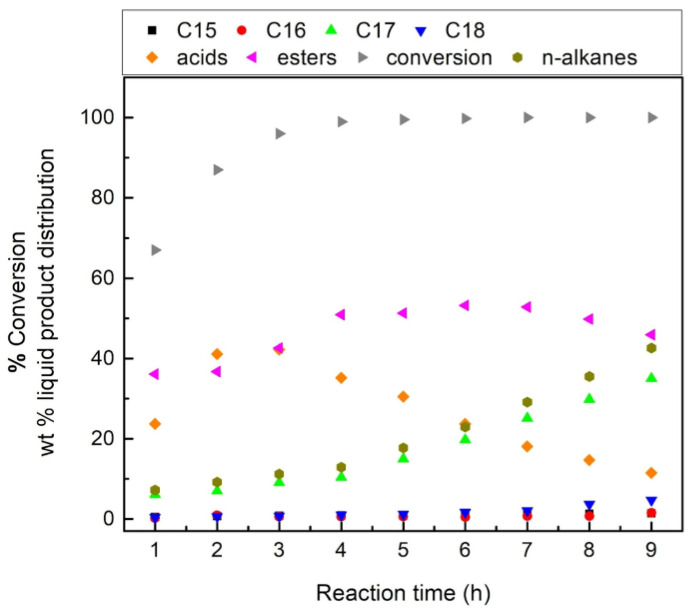
Kinetic curves of SDO obtained over the 60NiAl_cp(400)_ catalyst at reaction temperature 310 °C and H_2_ pressure 40 bar using 100 mL SFO and 1 g of catalyst.

Figure 7 illustrates kinetic curves obtained over the 60NiAl_cp(400)_ catalyst taken as a typical example.

An inspection of Figure 7 shows that the kinetic curves concerning the % composition of the liquid phase of the reactor in n-alkanes increase monotonically with time, indicating that these are the end products. In contrast, the kinetic curves concerning acids and esters pass through the maxima indicating that these are intermediate products. These findings are also in line with the SDO mechanism described in the Introduction.

Table 2 illustrates the percentage conversion of the feedstock obtained over the catalysts studied as well as the percentage composition of the liquid phase of the reactor in n-alkanes and the intermediate acids and esters for reaction time 9 h.

An inspection of this table clearly shows that the preparation method of catalysts affects the catalytic behavior. It can be seen that the composition of the liquid phase in n-alkanes, the most important evaluation parameter, is markedly higher over the catalyst prepared by co-precipitation (60NiAl_cp(400)_) than the catalyst prepared by wet impregnation (60NiAl_wi(400)_). Taking into account the fact that the active sites in SDO are developed on the nickel nanocrystals, the higher efficiency of the first catalyst can be effortlessly attributed to its higher specific surface area and smaller mean crystal size of nickel nanoparticles (thus ensuring a higher nickel metallic surface) with respect to the second catalyst (Table 1). Moreover, based on the aforementioned evaluation parameter, it can be seen that the increase in the activation temperature causes a decrease in the catalytic efficiency. This can be attributed to the increasing sintering of the nickel nanoparticles resulting in the increase in their mean crystal size and thus also the decrease in nickel metallic surface as well as to the blocking of small pores. This leads to a decrease in the specific surface area (Table 1) and a decrease in surface acidity (Figure 5). An inspection of Table 2 shows that the change in the preparation method and the activation temperature does not considerably influence the ratio [n-C_17_ + n-C_15_]/[n-C_18_ + n-C_16_] and thus the SDO network. In all cases, this ratio is much higher than unity, suggesting that SDO mainly proceeds via decarbonylation of the intermediate aldehydes.

### 3.3. Effect of the Reaction Temperature on the Performance of Most Efficient Catalyst Studied

**Figure 8:**
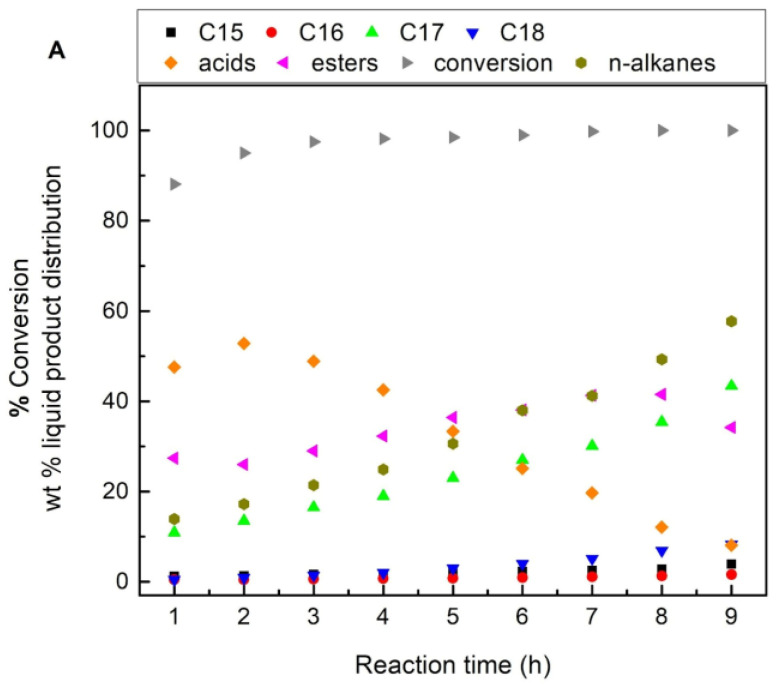

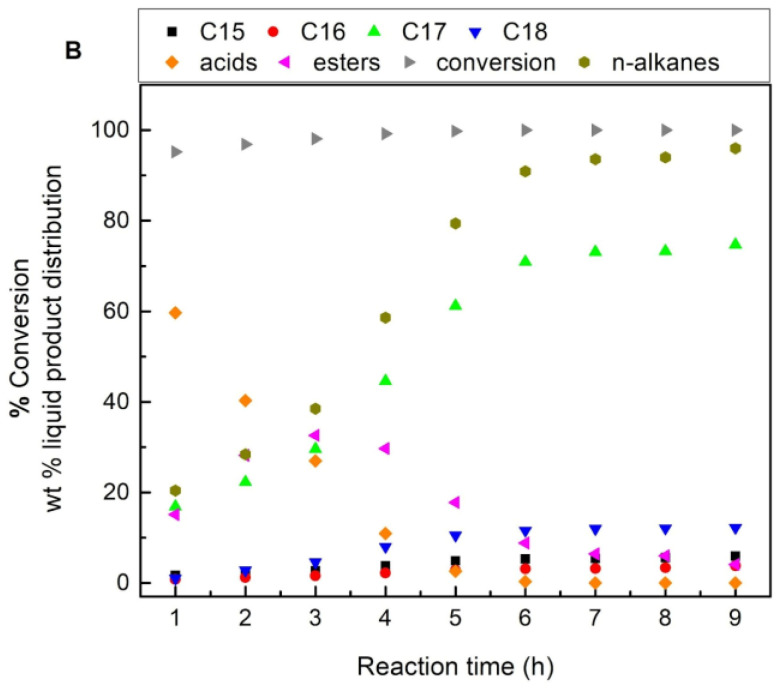
Kinetic curves of SDO obtained over the 60NiAl_cp(400)_ catalyst at reaction temperatures 330 °C (**A**) and 350 °C (**B**), H_2_ pressure 40 bar using 100 mL SFO and 1 g of catalyst.

Trying to optimize the catalytic performance of the most efficient, 60NiAl_cp(400)_ catalyst, we tested it at two higher temperatures (namely, 330 and 350 °C), keeping all the other reaction parameters as constants. Figure 8 illustrates the kinetic curves obtained. An inspection of this figure shows how the almost complete conversion of SFO was achieved after 3 h of reaction at 330 °C and 2 h at 350 °C instead of the 4 h needed at 310 °C (see Figure 7). Moreover, the green diesel obtained at 330 °C after 9 h of reaction represents 60% of the liquid reaction mixture instead of 42.6% obtained at 310 °C. This increased further at 350 °C, reaching 90% after 6 h and 97% after 9 h. The above clearly shows the impressive increase in the catalytic efficiency by increasing the reaction temperature from 310 °C to 350 °C. 

Kinetic studies are useful for the design of industrial processes (reactor design, optimization of production conditions) and for catalysts that are more efficient. They also contribute to a better understanding of the reaction mechanism. Such studies showed that the SDO mechanism involves the following steps: the first, very rapid step is the saturation of the unsaturated side chains of triglycerides. This is followed by the gradual decomposition of the O-C bonds on the glycerol side. This leads progressively to di-propyl esters, propyl esters, and propane. One molecule of fatty acid is released at each step. A small extent of decomposition of propyl esters to ethyl or methyl esters can be observed in some cases. The intermediate fatty acids are then reduced, leading to fatty aldehydes by releasing water molecules. The intermediate aldehydes are decarbonylated to n-alkanes. This pathway, called decarbonylation (deCO), is the main SDO route over nickel catalysts and leads to n-alkanes with one carbon atom less than the triglyceride chains. Alternatively, the intermediate aldehydes are reduced into the corresponding fatty alcohols. The latter are dehydrated to alkenes, which are then saturated. The resulting n-alkanes, through this pathway, called hydrodeoxygenation (HDO), have carbon atoms equal to the triglyceride chains. A third considerably less probable route over nickel catalysts is the direct decarboxylation (deCO_2_) of the intermediate fatty acids. In the SDO taking place under solvent-free conditions, the fatty acids may react with fatty alcohols resulting in high molecular weight esters, e.g., palmityl stearate and stearyl stearate (see Figure 6). The latter undergo hydrogenolysis to acids and n-alkanes and/or aldehydes and alcohols. The intermediate acids, aldehydes, and alcohols are then transformed into n-alkanes by SDO [7,20]. According to our results (Figure 7 and Figure 8), the SDO of the free fatty acids, and especially that of the aforementioned esters, are the slowest and thus produce rate-limiting steps. 

Lin et al. [39] recently reviewed kinetic studies concerning the SDO of vegetable oils. They found that two types of kinetic approaches have been adopted: one based on power law and another one, more detailed, adopting the Langmuir–Hinshelwood model. Kaluža and Kubička [40] have published a very interesting kinetic study modeling the aforementioned perplexing mechanism and adopting a simplified model of five pseudo-first-order reaction steps. In the present study, we are following a similar approach considering that the green diesel (n-alkanes) production obeys a pseudo-first-order reaction rate law [40,41]. Taking into account the results presented in Figure 7 and Figure 8, we calculated the values of the apparent reaction constant k concerning the hydrocarbon production at three reaction temperatures studied (310, 330, and 350 °C) using Equation (1).

**Figure 9:**
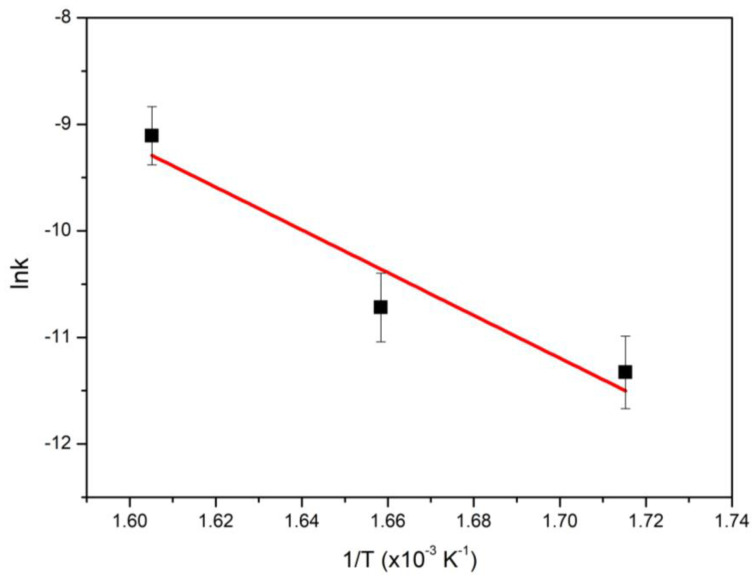
Arrhenius plot of apparent reaction constant (k) values versus temperature.

−ln(1−Y) = k × t,(1)

where Y is the yield of n-alkanes and t is the reaction time. Figure 9 shows that when these values are introduced in an Arrhenius plot, a very good linear correlation is obtained. This indicates that our assumption for the reaction rate law is reasonable. From the slope of the straight line, we calculated an apparent activation energy E_α_ = 83 kJ mol^−1^, which corresponded to the rate-limiting steps (SDO of free fatty acids and high molecular weight esters). This value is in the range of E_α_ values for chemical reactions indicating that mass transfer phenomena do not affect our results.

The E_α_ value determined in the present study is between those calculated by Anand et al. [42,43] for green diesel production using CoMo/Al_2_O_3_ (47 kJ mol^−1^) and NiW/Al_2_O_3_-SiO_2_ (115 kJ mol^−1^) sulfided catalysts.

**Figure 10:**
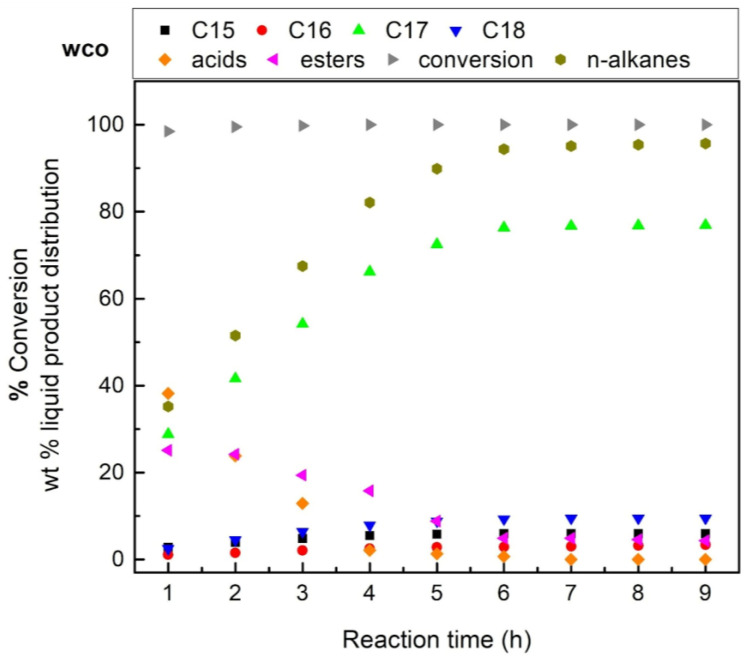
Kinetic curves of SDO obtained over the 60NiAl_cp(400)_ catalyst at reaction temperatures 350 °C, H_2_ pressure 40 bar, using 100 mL WCO and 1 g of catalyst.

Taking into account the social debate concerning the conflict between food and first-generation biofuel production, we tested the most efficient 60NiAl_cp(400)_ catalyst in the transformation of waste cooking oil (WCO) to renewable diesel. The test was performed at a reaction temperature of 350 °C and H_2_ pressure of 40 bar, using 100 mL WCO and 1 g of catalyst. Figure 10 shows the kinetic results obtained.

Comparing the results depicted in Figure 10 with those of Figure 8B, one can conclude that the 60NiAl_cp(400)_ catalyst works equally well for green diesel production from WCO and SFO.

### 3.4. Influence of Reaction Temperature and Feedstock on Spent Catalyst Characteristics

Spent 60NiAl_cp(400)_ catalyst samples obtained after catalytic tests for 9 h at three reaction temperatures (310, 330, and 350 °C) were washed with hexane in a microwave bath several times in order to remove organic molecules adsorbed on the catalyst surface or trapped inside the catalyst pores. These samples were characterized by XRD, N_2_-physisorption, and high-temperature combustion analysis. Table 3 compiles the results obtained.

XRD analysis of the spent catalyst samples (not presented) show that the supported nickel exists exclusively in its metallic form (Ni^0^) after the reaction independently of the temperature used. This means that the NiO detected in the activated fresh catalyst (Figure 3) was completely reduced upon reaction at high H_2_ pressure (positive effect). However, this reduction was accompanied by the sintering of Ni^0^, which led to a gradual increase in Ni^0^ nanoparticles with a reaction temperature (negative effect, Table 3). Testing the catalyst at 310 °C with SFO as the feedstock, the sintering was combined with another negative effect: the enhanced coke formation (19.71% C). The combination of these two negative effects resulted in a dramatic decrease in the spent catalyst S_BET_ (10 times) and SPV_BJH_ (3 times) values, as well as an increase in the MPD_BJH_ value (Table 3). These results show that the aforementioned changes affected mainly the narrow catalyst pores. However, the increase in the reaction temperature provoked a decrease in the coke deposited on the catalyst surface (positive effect). The decrease in catalyst propensity for coke formation as the reaction temperature increased has been observed several times for nickel catalysts [44,45]. This decrease is reflected in the decreasing changes of the S_BET_, SPV_BJH,_ and MPD_BJH_ values with respect to the corresponding ones obtained in the fresh catalyst (Table 3).

The use of WCO as feedstock led to a significantly increased coke formation (9.40% C in the case of WCO instead of 5.01% C in the case of SFO at a reaction temperature of 350 °C), affecting more intensively the textural characteristics of the catalyst (Table 3). This finding can be attributed to various bulky organic impurities (e.g., starch and proteins) being present in the WCO [22].

Closing this chapter, we have to notice that the high efficiency exhibited by the 60NiAl_cp(400)_ catalyst at a reaction temperature of 350 °C shows that the decrease in coke formation as the reaction temperature increases overcompensates for the negative effects mentioned. However, the reusability of this catalyst is an open research issue that is now under study in our lab.

### 3.5. Comparison of Catalyst Efficiency for Green Diesel Production with Relevant Catalysts

The nickel—alumina catalyst prepared by co-precipitation (60NiAl_cp(400)_) in the present work showed that this preparation method leads to very promising SDO catalysts. This can be supported by comparing the efficiency of the above catalyst (96–97% green diesel yield at 350 °C) with catalysts that have appeared in the recent literature. To be more specific, Nugrahaningtyas et al. [46] prepared a nickel/mordenite catalyst following the wet impregnation method combined with two reflux steps and evaluated these catalysts for the hydrotreatment of WCO, achieving a 56.6% green diesel yield at 350 °C. Jeon et al. [47] studied the deoxygenation of oleic acid for green diesel production over Ni/MgO-Al_2_O_3_ catalysts prepared by incipient wetness impregnation. They found that a catalyst containing 20 wt. % nickel proved the most efficient leading to a 44.4% green diesel yield at a reaction temperature of 300 °C. Zafeiropoulos et al. [30] studied Ni/SiO_2_ catalysts with various Ni loadings. Their most efficient catalyst led to a 67% green diesel yield at 310 °C, containing 50 wt. % Ni, and was prepared by wet impregnation. Prihadiyono et al. [48] prepared Ni catalysts supported on Indonesian natural zeolite for transforming crude palm oil into green diesel. Their most active catalyst succeeded in a 77.34 % green diesel yield at a reaction temperature of 375 °C. Sowe et al. [49] prepared nickel catalysts (15 wt. % Ni) supported on mixed oxide supports (Al_2_O_3_–ZrO_2_ and SiO_2_–ZrO_2_) and tested them in the hydrodeoxygenation of hydrolyzed WCO at 350 °C. High green diesel yields that were (90.5–97.1%) rich in pentadecane were obtained over these catalysts. 

## 4. Conclusions

A nickel-alumina catalyst containing 60% Ni, prepared by co-precipitation and activated at 400 °C, 60NiAl_cp(400)_, exhibited a higher specific surface area, a smaller mean crystal size of the nickel nanoparticle and thus higher nickel metallic surface, which justifies its higher efficiency with respect to the corresponding catalyst synthesized by wet impregnation, 60NiAl_wi(400)_. The increase in the activation temperature from 400 up to 600 °C increased the size of the nickel nanoparticles through sintering and thus destroyed the small pores. This led to the decrease in the nickel surface and specific surface area and thus to the decrease in catalytic efficiency. The increase in the reaction temperature positively affected the catalyst performance of the most active catalyst, 60NiAl_cp(400)_, diminishing the coke formation, although this led the catalyst’s textural characteristics to be downgraded. The study showed that an almost complete transformation of SFO and WCO into green diesel was obtained at 350 °C. In fact, the liquid product obtained after 7 h of reaction contained 96–97% n-alkanes using SFO or WCO as feedstock.

## Figures and Tables

**Table 1:** Physicochemical characteristics of the catalysts studied.

Supports/Catalysts	S_BET_ m^2^·g^−1^	^1^ SPV_BJH_ cm^3^·g^−1^	^2^ MPD_BJH_ nm	^3^ MCD_Ni_ nm	^4^ S_Ni_ m^2^·g^−1^	^5^ CO_chem_ μmol/g_cat_	^6^ Acidity a.u.
γ-Al_2_O_3_	260	0.66	6.6	-	-	-	-
0NiAl_cp(400)_	344	0.34	3.2	-	-	-	-
60NiAl_w.i(400)_	111	0.29	7.6	20.2	20.0	7	14
60NiAl_cp(400)_	272	0.60	6.6	<4.0	>60	15 ^7^	63
60NiA_cp(500)_	193	0.59	8.7	7.6	53.2	10.5	57
60NiAl_cp(600)_	143	0.54	10.9	8.2	49.3	9	54

^1.^ SPV_BJH_: specific pore volume, ^2.^ MPD_BJH_: mean pore diameter. The BJH methodology concerns pores in the range 1.7–300 nm. ^3.^ MCD_Ni_: mean crystal diameter of metallic nickel determined on the base of the peak at 2θ = 51.8°. ^4.^ Surface of metallic nickel calculated on the base of MCD_Ni_. ^5.^ CO chemisorbed on Ni^0^ surface measured by pulse technique. ^6^ Amount of desorbed NH_3_ calculated by the area under TPD curves. ^7^ Value taken from Ref. [22].

**Table 2:** Percentage of the feedstock conversion (X) composition of the liquid phase in n-alkanes, acids, and esters (wt. %) and odd to even carbon atoms ratio of hydrocarbons (R) obtained over the nickel catalysts studied for the SDO of SFO (310 °C, 40 bar H_2_ pressure, 100 mL/min H_2_ flow, 100 mL of SFO/g of catalyst and 9 h of reaction time).

Catalyst	X (%)	Liquid Phase Composition (wt. %)							R *
		Acids	Esters	Alkanes	n-C_17_	n-C_18_	n-C_15_	n-C_16_	
60NiAlwi(400)	78.8	9.7	51.0	18.2	14.3	2.7	0.9	0.3	5.1
60NiAlcp(400)	100	11.5	45.9	42.6	35.0	4.7	1.4	1.5	7.5
60NiAlcp(500)	95.1	10.2	61.3	23.6	19.8	2.0	1.4	0.4	8.8
60NiAlcp(600)	93.2	12.0	60.6	20.7	17.1	1.9	1.2	0.4	8.0

* [n-C_17_+n-C_15_]/[n-C_18_+n-C_16_] ratio.

**Table 3:** Specific surface area (S_BET_), specific pore volume (SPV_BJH_), mean pore diameter (MPD_BJH_), mean size of Ni^0^ crystals (MCD_Ni_), and coke content of spent 60NiAl_cp(400)_ catalyst tested at 310, 330 and 350 °C.

Reaction Temperature °C	S_BET_ m^2^g^−1^	SPV_BJH_ cm^3^g^−1^	MPD_BJH_ nm	MCD_Ni_ nm	Carbon Content %
- *	272	0.60	6.6	<4	--
310	27	0.19	20.4	10.2	19.71
330	73	0.18	16.1	14.0	7.40
350	110	0.52	13.5	20.9	5.01
350 **	52	0.3	15.6	19.2	9.40

* The corresponding values for the fresh 60NiAl_cp(400)_ catalyst are included for comparison. ** Using waste cooking oil as feedstock.

## Data Availability

All data supporting the reported results can be found in the published article.

## Supplementary Materials

The following supporting information can be downloaded at: https://www.mdpi.com/article/10.3390/nano13030616/s1, Figure S1: SEM images of A) 60NiAl_wi(400)_ and B) 60NiAl_cp(400)_; Figure S2: N_2_ adsorption-desorption isotherms of alumina supports (γ-Al_2_O_3_ and 0NiAl_cp(400)_) and nickel-alumina catalysts (60NiAl_wi(400)_ and 60NiAl_cp(400)_); Figure S3: N_2_ adsorption-desorption isotherms nickel-alumina catalysts activated at various temperatures (400, 500 and 600 °C); Figure S4: XRD patterns of the alumina samples (0NiAl_wi(400)_: commercial support used for the preparation of Ni catalyst by wet impregnation; 0NiAl_cp(400)_: prepared following the co-precipitation method without addition of Ni(NO_3_)_2_ 6H_2_O in the initial solution).
