# Supplementary material for: Nickel—Alumina Catalysts for the Transformation of Vegetable Oils into Green Diesel: The Role of Preparation Method, Activation Temperature, and Reaction Conditions
Source: Nanomaterials (Basel). 2023 Feb 3;13(3):616. doi: 10.3390/nano13030616 (PMC9919930; doi:10.3390/nano13030616)
Supplement: Supplementary file 1 [file nanomaterials-13-00616-s001.zip › nanomaterials-2143767-supplementary.pdf]

Supplementary material

Nickel – alumina catalysts for the transformation of vegetable oils into green diesel: the role of preparation method, activation temperature and reaction conditions

Ioannis Nikolopoulos[1], George Kogkos[1], Vasiliki D. Tsavatopoulou[1], Eleana Kordouli[1,2], Kyriakos Bourikas[2], Christos Kordulis[1,2,3]* and Alexis Lycourghiotis[1]

[1]Department of Chemistry, University of Patras, GR-26504, Patras, Greece
[2]Hellenic Open University, Parodos Aristotelous 18, GR-26335, Patras, Greece
[3]Foundation for Research and Technology, Institute of Chemical Engineering Science (FORTH/ICE-HT), Stadiou Str., Platani, P.O. Box 1414, GR-26500, Patras, Greece
*Correspondence: kordulis@upatras.gr

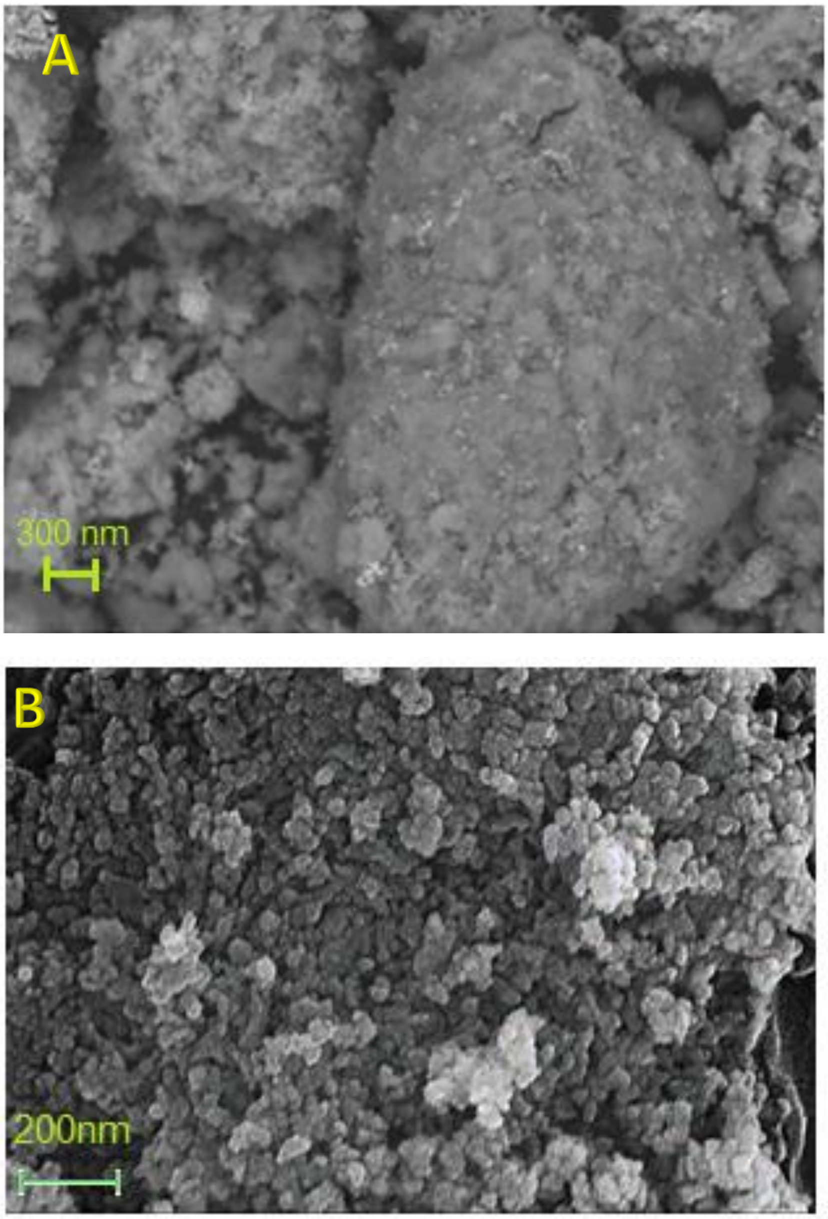

Figure S1: SEM images of A) $60NiAl_{wi(400)}$ and B) $60NiAl_{cp(400)}$.

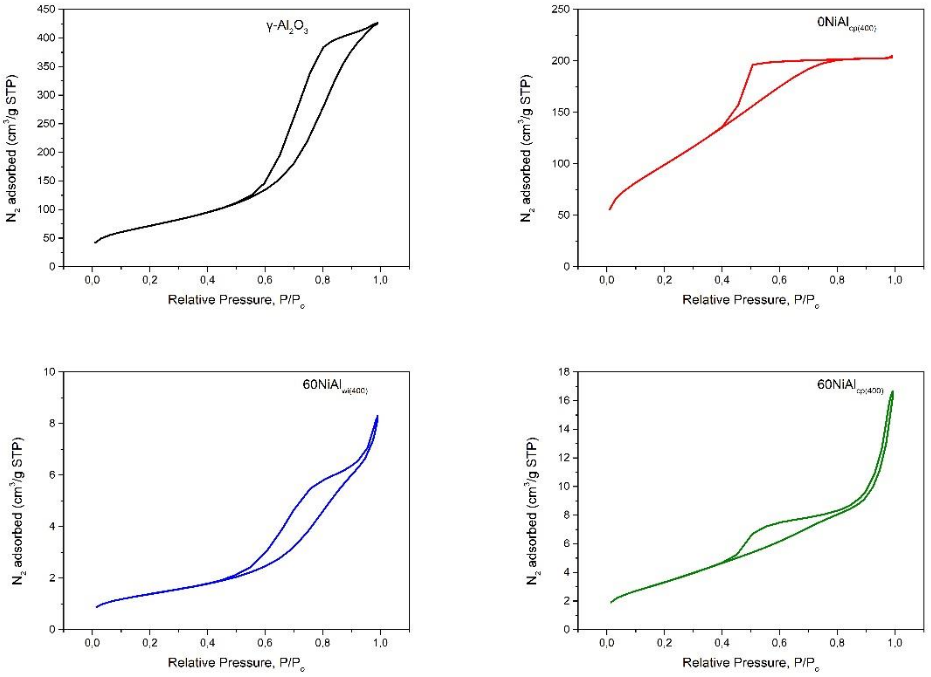

Figure S2: N_2 adsorption-desorption isotherms of alumina supports (γ-Al_2O_3 and $0NiAl_{cp(400)}$) and nickel – alumina catalysts ($60NiAl_{wi(400)}$ and $60NiAl_{cp(400)}$).

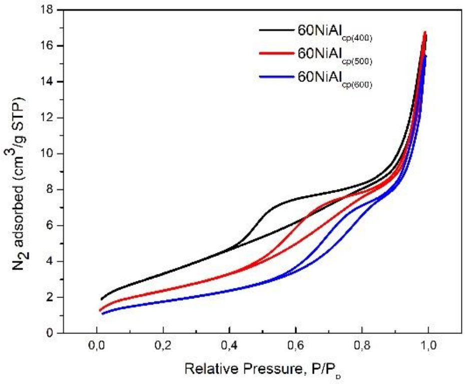

Figure S3: N_2 adsorption-desorption isotherms nickel – alumina catalysts activated at various temperatures (400, 500 and 600 °C).
